# Do the Bugs in Your Gut Eat Your Memories? Relationship between Gut Microbiota and Alzheimer’s Disease

**DOI:** 10.3390/brainsci10110814

**Published:** 2020-11-03

**Authors:** Emily M. Borsom, Keehoon Lee, Emily K. Cope

**Affiliations:** The Pathogen and Microbiome Institute, Center for Applied Microbiome Science, Northern Arizona University, Flagstaff, AZ 86011, USA; emb366@nau.edu (E.M.B.); Keehoon.lee@nau.edu (K.L.)

**Keywords:** microbiome, gut microbiota–brain axis, Alzheimer’s disease, neuroinflammation, microglia, astrocytes

## Abstract

The human microbiota is composed of trillions of microbial cells inhabiting the oral cavity, skin, gastrointestinal (GI) tract, airways, and reproductive organs. The gut microbiota is composed of dynamic communities of microorganisms that communicate bidirectionally with the brain via cytokines, neurotransmitters, hormones, and secondary metabolites, known as the gut microbiota–brain axis. The gut microbiota–brain axis is suspected to be involved in the development of neurological diseases, including Alzheimer’s disease (AD), Parkinson’s disease, and Autism Spectrum Disorder. AD is an irreversible, neurodegenerative disease of the central nervous system (CNS), characterized by amyloid-β plaques, neurofibrillary tangles, and neuroinflammation. Microglia and astrocytes, the resident immune cells of the CNS, play an integral role in AD development, as neuroinflammation is a driving factor of disease severity. The gut microbiota–brain axis is a novel target for Alzheimer’s disease therapeutics to modulate critical neuroimmune and metabolic pathways. Potential therapeutics include probiotics, prebiotics, fecal microbiota transplantation, and dietary intervention. This review summarizes our current understanding of the role of the gut microbiota–brain axis and neuroinflammation in the onset and development of Alzheimer’s disease, limitations of current research, and potential for gut microbiota–brain axis targeted therapies.

## 1. Introduction 

The human microbiota, the aggregate of all bacterial, viral, fungal, and archaeal cells that inhabit the human body, consists of 1–1.5× more microbial cells than human cells (~10^14^) [1]. The microbiome, or the collective genomes of the resident microbes, resides throughout the entire human body, including the skin, oral cavity, respiratory tracts, vaginal cavity, and the gastrointestinal (GI) tract [2]. Recent studies of the human microbiome demonstrate a myriad of roles that these microbes play in host health, including host immune function [3,4], protection against pathogen colonization [5], and host metabolism [6]. Perturbations to normal gut microbiome functions are associated with a variety of diseases, including GI, autoimmune, neurological, and metabolic diseases [7]. The GI tract houses about 70% of the human microbiota and comprises Firmicutes, Bacteroidetes, Actinobacteria, Proteobacteria, and Verrucomicrobia [8]. These microbes aid in digestion [6], vitamin synthesis [9], and development of the nervous, endocrine, and immune system [3,4]. Members of the genera *Bacteroides, Prevotella*, and *Lactobacillus* produce B vitamins which play important roles in the host immune regulation [10]. The development of the immune system is influenced by the gut microbiome, for instance, a study by Atarashi et al. demonstrated that oral inoculation of *Clostridium* strains in germ-free mice induced colonic CD4+ T regulatory cells and reduced colitis [11]. Gut microbes are able to ferment indigestible carbohydrates, producing short chain fatty acids (SCFA) as a byproduct, and thereby promote maturation of the immune system, including colonic regulatory T cells [12] and bone marrow hematopoiesis of new circulating monocytes and dendritic cells [13,14]. In the GI tract, dysbiosis of the microbial communities can lead to overgrowth of pathogenic bacteria and a decrease in the integrity of the intestinal barrier, allowing proinflammatory molecules to circulate throughout the bloodstream [15]. For example, *Ruminococcus gnavus*, a common gut pathogen in Crohn’s disease patients, utilizes mucin as a carbon source, directly breaking down the gut mucosal barrier and producing proinflammatory cytokines, including Tumor necrosis factor (TNF)-α [16].

The endocrine, neural, metabolic, and immune mechanisms that comprise the gut microbiota–brain axis contribute significantly to overall host health [17]. For instance, decreased alpha diversity in humans is correlated with decreased systemic estrogens. Depleted systemic estrogens are linked to cognitive decline, memory loss, and reduced fine motor skills [18,19]. Another key player in the gut microbiota–brain axis is the vagus nerve, which allows for direct communication via neurotransmitters between the CNS and the enteric nervous system (ENS) [20]. As an example, the gut bacteria *Lactobacillus rhamnosus* can regulate GABA receptors in mice, therefore reducing depressive behaviors via the vagus nerve [21]. Severing the vagus nerve reversed this outcome, negating the beneficial effects of *L. rhamnosus* in mitigating depressive behavior. Additionally, secondary metabolites and cytokines produced by the host–microbial interactions in the gut can cross the intestinal barrier and travel through the blood to induce systemic inflammation [2]. One study found increased levels of the plasma cytokines Interleukin (IL)-2, IL-1β, and Interferon (IFN)-γ in Ldlr−/− mice, an atherosclerosis mouse model, which were colonized with the proinflammatory gut microbiota from Casp1−/− mice, a knockout inflammatory mouse model, via fecal microbiota transplants. These findings suggest that the dysbiotic gut microbiota of the Casp1−/− mice shifted the gut microbiota of FMT treated Ldlr−/− mice. This shift led to a decrease in short chain fatty acids (SCFA) and an increase in the nuclear factor kappa-B (NF-κB) activity in immune cells, thereby increasing the production of proinflammatory cytokines [2,22]. 

The role of the gut microbiota–brain axis has been implicated in many neurological disorders and diseases, including multiple sclerosis, gliomas, Parkinson’s disease, and Alzheimer’s disease (AD) [23]. In non-AD dementia, there was a lower relative abundance of *Bacteroides* compared to the gut microbiome of cognitively non-impaired subjects, which is the opposite pattern of what is observed in the AD gut microbiome [24]. Another study analyzed and compared the fecal microbiome and metabolites of patients with and without dementia, and the gut microbiota-associated metabolite of dementia patients was distinct from non-dementia patients [25]. For example, high phenol and p-cresol were observed in patients with dementia, which induces an increase in fecal ammonia and increases the risk of dementia. Conversely, in people without dementia, high fecal lactic acid was observed which was associated with a low risk of dementia [25]. Vascular cognitive impairment (VCI), a common cause of dementia, may be mediated with metabolites produced by gut microbiota. For example, lipopolysaccharide (LPS) and trimethylamine-N-oxide (TMAO) from gut microbiota can contribute to the increased intestinal epithelial permeability that leads to immune responses associated with VCI [26].

Several studies have demonstrated that celiac disease (CD) is associated with neurological disease development [27,28]. CD is an autoimmune disease caused by inability to digest gluten in the small intestine. Due to intestinal epithelial dysfunction caused by CD, immunotoxic gluten peptide is introduced into the circulatory system and passes through the BBB, causing neuroinflammation. CD also decreases the expression of PPAR-γ, which plays a role in modulating inflammation. Decreased PPAR-γ is associated with dysbiosis of the gut microbiome and an increase in factors related to AD development such as elevated LPS, pro-inflammatory cytokines, and bacterial metabolites. One study demonstrated that CD-induced changes in the gut microbiome and neurological disease development can be improved through a gluten-free diet (GFD). Not only did GFD reduce the amount of neuroinflammation, but it also correlated with an increase in the expression of PPAR-γ and gut microbiome health [27]. In addition, Transcranial magnetic stimulation (TMS), a non-invasive brain stimulation technique, enables early diagnosis of “hyperexcitable celiac brain” that often appears before dementia in CD patients even before any onset of clear symptoms, so that these patients can be prescribed a GFD to prevent neurodegeneration that can lead to dementia as early as possible [29,30].

In this review, we present an up-to-date, comprehensive evaluation of the field of gut microbiome research in Alzheimer’s disease, with a unique perspective on the potential role of the gut microbiome in neuroinflammation. We also summarize a potential mechanistic pathway describing a hypothesized role for LPS in AD pathologies. As Hippocrates once said, “All disease begins in the gut” [31]. Could this statement hold the truth, even after 2500 years? In this review, we will discuss the current understanding of the role of the gut microbiota–brain axis in AD and provide commentary on potential mechanisms for AD pathogenesis. 

### Methodology

Search terms to identify appropriate literature for this review using Google Scholar include, but are not limited to, “Alzheimer’s disease”, “gut microbiota”, “gut microbiota–brain axis”, in combination with “microglia”, “astrocytes”, “LPS”, and “neuroinflammation”. This review provides a current, comprehensive overview of the gut microbiota brain axis in AD, with emphasis on modern molecular techniques, gut microbiota-induced neuroimmune pathways, and uncovering potential therapeutics. Primary studies that led to novel insights on the gut microbiome and AD in preclinical murine models and human AD participants were included in this literature review.

## 2. Characterization of Alzheimer’s Disease

AD was first identified by psychiatrist and neuropathologist, Alois Alzheimer, in 1906. After observing plaques and neurofibrillary tangles in the brain histology of a patient who suffered from memory loss, aggression, confusion, and paranoia, he presented his findings at the 37th Meeting of South-West German Psychiatrists. Shortly after, the disease characterized by Alzheimer was coined with the name “Alzheimer’s disease” by his colleague, Emil Kraepelin [32]. Today, Alzheimer’s disease (AD) is the most common cause of dementia, affecting 5.8 million people in the United States alone. The prevalence of Alzheimer’s disease is predicted to rise to 13.8 million in the United States by 2050 [33]. Apolipoprotein E (*APOE*) genotype is recognized as the strongest genetic risk factor for the development of AD. The *APOE* protein is involved in several biochemical regulatory processes, including immunoregulation, neuroinflammation, and neuroprotection. *APOE* exists in three isoforms, although *APOE4* prevails as the strongest predictor of dementia. The prevailing hypothesis is the cascade hypothesis, which states that amyloid-β plaque deposition leads to intracellular neurofibrillary tangles caused by hyperphosphorylation of the protein tau and disintegration of the microtubules in neurons, resulting in loss of neuronal function [34]. These two hallmark pathologies, extracellular amyloid-β plaques and intracellular neurofibrillary tau tangles, are present in the hippocampus and prefrontal cortex, and result in loss of neuronal function and cognitive impairment [35]. Amyloid plaques form when the amyloid precursor protein (APP) is sequentially cleaved by endogenous proteases, β-protease and γ-protease. This cleavage event renders peptides ranging in length and solubility, yet Aβ-42 tends to form soluble fibrils, and is the predominant species observed in the brain of severe AD patients [36,37]. Amyloidosis may begin as early as 10–20 years before cognitive decline or AD symptoms become apparent [38]. In addition to plaques and tangles, neuroinflammation is increasingly recognized as central to disease progression; current studies have identified both microglia and astrocyte induced inflammation as a key pathological feature of AD [39]. While not yet considered a core pathology for diagnosis, neuroinflammation remains a driving feature in AD pathogenesis [40]. 

## 3. Techniques for Microbiome Analysis

Advances in the fields of sequencing and bioinformatics allow for increasingly accurate analysis of microbial communities and their role in human health. The field of microbiome sequencing has been dominated by marker gene sequencing. For bacteria, 16S rRNA gene is a widely used marker gene, and fungi can be identified using marker sequences on the 18S rRNA gene or the ITS (internal transcribed spacer) region. Selection of the 16S rRNA gene was based on the presence of nine highly variable regions within the gene, allowing for bacterial taxonomic classification, that are flanked by conserved regions that allow for primer design [41]. The ITS regions are situated between the 18S rRNA gene and the 5.8S rRNA gene (ITS1) and the 5.8S rRNA gene and the 28S rRNA gene (ITS2). ITS1 and ITS2 have high evolutionary rates, allowing for greater taxonomic resolution than is possible with 18S rRNA gene sequencing [42]. However, for those who are interested in species- and strain-level taxonomic resolution, shallow shotgun metagenomic sequencing (SSMS) is becoming increasingly popular [43]. While SSMA does not provide the depth of taxonomic and functional resolution that deep shotgun metagenomic sequencing does, it is relatively low cost compared to deep shotgun metagenomics, and therefore more feasible to apply in large-scale studies [43]. 

Functional and mechanistic aspects of the microbiome’s integral role in health or disease can be assessed by integrating a multi’omics approach including deep shotgun metagenomics, transcriptomics, and metabolomics. As discussed above, deep shotgun metagenomic sequencing will provide strain-level and functional information encoded in the DNA content of a microbiome. In shotgun metagenomics, all of the DNA present in a sample is sequenced with minimal PCR amplification bias. This allows characterization of the species and encoded genes in a wide array of microorganisms, including fungi, bacteria, archaea, parasites, and DNA viruses. Although metagenomic sequencing can characterize the functional potential of a microbial community, this DNA-based approach is unable to determine differences in gene expression in a given environment. Transcriptomics, or RNA-seq, can be used to evaluate changes in gene expression. Finally, we can identify and quantify primary and secondary metabolites (e.g., short chain fatty acids) produced by the host and microbiota using metabolomics. Metabolomics can lead to a better understanding of microbial communication and microbial community involvement in metabolic pathways [44]. Understanding community composition is essential for microbiome studies; however, an integrated, multi-omics approach is critical to elucidate the microbial taxa and mechanisms essential to host health and disease. 

## 4. Gut Microbiota–Brain Axis in Alzheimer’s Disease

Although amyloid plaques and tau tangles are thought to be central to AD, over 2000 clinical trials targeting plaques, tangles, neurotransmitters, and other related mechanisms have failed to successfully treat AD [45]. However, the bidirectional communication between the GI tract and the central nervous system (CNS) via immune, endocrine, neural, and metabolic pathways, known collectively as the gut microbiota–brain axis, has recently been hypothesized to contribute to AD etiology and pathogenesis [23,46]. The human gut microbiome influences neuroinflammation in AD through the production of proinflammatory cytokines and bacterial metabolites that can enter circulation and reach the brain to act on neuronal immune cell populations [47]. Proinflammatory cytokines involved in AD pathogenesis include IL-1β, IL-6, IL-18, TNF-α, and IFN-γ [48]. Overexpression of IL-1 has been shown to favor plaque deposition in vivo and in vitro [49], while upregulation of IL-6 is associated with hyperphosphorylation of tau, leading to neuronal degradation [49,50]. Both Aβ plaques and neurofibrillary tangles are co-localized with activated glial cells in the CNS, suggesting gliosis plays a major role in AD pathogenesis and neuroinflammation [51]. In addition to cytokines, bacterial metabolites, including trimethylamine *N*-oxide (TMAO) and SCFAs may play a role in the development of or protection against AD pathologies. TMAO, a gut microbiome-produced metabolite previously linked to cardiovascular disease [52], is increased in the cerebrospinal fluid of AD patients and is directly correlated to CSF tau biomarkers, suggesting a role in brain aging and cognitive decline [53]. On the other hand, SCFAs, particularly valeric, butyric, and propionic acid, have the potential to ameliorate amyloidosis by interfering with Aβ1-40 and Aβ1-42 peptide interactions, thereby preventing conversion to neurotoxic Aβ plaque formation [54]. We expand on each of these topics, below. 

### Enteric Nervous System: Vagus Nerve

The enteric nervous system (ENS) is the largest part of the autonomic nervous system, composed of over 100 trillion neurons that function independently from the CNS [55]. The neural circuits that make up the ENS control local motor function and blood flow, fluid secretions and transports, and regulate immune and endocrine functions [56]. As a major component of the gut microbiota–brain axis, the vagus nerve directly connects the gut to the brain and spinal cord [17]. The vagus nerve mediates signaling pathways for satiety, stress, and mood via microbial and neural signaling [57]. Therefore, perturbations to the nerve disturb the gut–microbiota brain axis, leading to gastrointestinal diseases, including irritable bowel syndrome [58]. However, the vagus nerve is also able to receive input from the gut microbiota and transfer this information to the central nervous system [59]. To illustrate, enteroendocrine cells (EECs), making up 1% of gut epithelial cells, release 5-HT (serotonin precursor) in response to chemical or mechanical stimulation, which stimulates 5-HT3 receptors on the vagus nerve. Stimulation of these receptors controls physiological responses including peristalsis, gut motility, and other visceral functions [60]. 

In terms of treatment options, the vagus nerve remains a viable target for AD treatment. Clinical trials targeting the vagus nerve through stimulation have shown some cognitive improvement in AD patients up to one year post-treatment [61,62]. Vagus nerve stimulation (VNS) has also shown sustained, long term cognitive improvement in refractory depression patients [63]. A more recent study in transgenic APP/PS1 mice reversed morphological signs of aging and activation in 12 month mice using non-invasive vagus nerve stimulation [64]. However, the use of VNS as a therapeutic for AD is still a novel idea and requires further investigation of the mechanisms involved.

## 5. Gut Microbiota Composition and Diversity in Individuals with AD

The hypothesis that members of the gut microbiota contribute to AD onset and progression via the gut microbiota–brain axis has emerged in the past five years. However, few studies have been completed in the clinical setting. To our knowledge, five studies have characterized the gut microbiota composition in human AD patients compared to age-matched, non-AD controls, all showing an altered bacterial microbiota composition in AD patients. A cross sectional study of the gut microbiota composition in patients selected for their *APOE* genotype demonstrated that *APOE4* carriers had decreased abundance of butyrate producing gut bacteria, including *Clostridium*, and lower levels of fecal SCFAs compared to other *APOE* genotypes, indicating a relationship between *APOE* isoforms and gut microbiota compositions [65]. The second study conducted in AD patients demonstrated a decrease in the phyla Firmicutes and an increase in Bacteroidetes, as well as an increase in the genus *Blautia* in AD patients. They also observed decreased alpha (within sample) diversity in AD patients. Additionally, they observed correlations between CSF markers of AD and relative abundance of taxa; a significant positive correlation was demonstrated between the genus *Bacteroides* and CSF YK-40, a marker of astroglia and microglia activation. These findings suggest an increase in *Bacteroides* may be linked to increased neuroinflammation [66]. We discuss the potential mechanisms below. The other three studies demonstrate a gut microbiota composition with decreased abundance of SCFA-producing bacteria and increased abundance of proinflammatory bacteria when comparing AD patients to non-dementia, aged-matched controls [67,68,69]. Taxa identified in the individual studies can be found in Table 1. 

Alterations in the bacterial gut microbiota have been observed in AD patients, indicating a potential role for gut microbes in AD. Three of the five studies in human patients showed significant changes in the genus *Bacteroides*, although the directionality of this change, whether *Bacteroides* relative abundance increased or decreased, differed (Table 1). Future research to determine whether environmental factors independent of AD pathologies cause the observed shifts in the gut microbiome are necessary. For example, dietary changes as a person progresses through dementia, the influence of medications on the composition of the gut microbiota in AD, decreased body mass, and overall well-being should be considered as potential mediators of the gut microbiota composition and diversity. Finally, changes in the microbiota may extend beyond the gut. The post mortem brain of AD patients has a higher bacterial burden when compared to age matched controls, consisting mostly of Actinobacteria, particularly *Propionibacterium acnes* [70]. Additionally, mice infected orally with *Porphyromonas gingivalis* exhibited brain colonization associated with increased amyloidosis [71]. These findings provide intriguing evidence for translocation of microbes from the gut, suggesting that microbes have the ability to cross both a dysfunctional gut epithelial barrier and blood brain barrier. Further studies are required to determine whether microbial translocation from other body sites to the brain occurs in AD. 

## 6. Gut Microbiota Composition and Diversity in Murine Models of Key AD Pathologies

Transgenic mouse models of key AD pathologies are an essential tool in mechanistic studies and drug discovery with the potential for translational treatments in clinical trials [32]. Numerous studies have shown that transgenic murine models have unique gut microbiota compositions when compared to the gut microbiota compositions of wild-type mice of the same genetic background or compared to transgenic mice with the wild-type allele [35,72,73]. Parikh and colleagues assessed changes in the gut microbiota composition of 5xFAD mice, a transgenic familial model of plaque deposition that was homozygous for *APOE2*, *APOE3*, or *APOE4*. Significant changes observed in the gut microbiota between genotypes regardless of 5xFAD mutations suggests a relationship between the gut microbiota composition and *APOE* isoforms [74]. In a study by Chen and colleagues, transgenic APP/PS1 mice, a model bearing the mutations in transgenes for APP and PSEN1 resulting in Aβ plaque deposition, demonstrated altered microbiota preceding amyloidosis and microgliosis, suggesting a role of the gut microbiota in AD pathogenesis (Table 1, [72]). In 5xFAD mice, which express human APP and PSEN1 transgenes with five AD-linked mutations, leading to rapid development of amyloidosis, an increase in the phylum Firmicutes and a decrease in the phylum Bacteroidetes was observed at nine weeks. More specifically, *Clostridium leptum* was increased in 5xFAD mice, a common gut bacterium associated with gut inflammation [73]. When raised germ-free, APP/PS1 mice exhibited significantly reduced amyloidosis and microgliosis, suggesting that in absence of the gut microbiota, AD pathologies are less severe and supports the hypothesis that the gut microbiota plays a critical role in AD pathologies [75].

To better understand the mechanisms behind these alterations in the gut microbiota composition of transgenic models of AD pathologies, including amyloid deposition and neurofibrillary tangles, manipulation of the gut microbiota allows for uncovering potential processes involved in AD development. Manipulation of the gut microbiota through prebiotics, probiotics, and fecal microbiota transplants are potential therapeutic options for AD and have been explored in preclinical murine models of AD pathologies (Table 2). Bonfili and colleagues administered SLAB51, a mixture of nine live bacterial strains, to 3xTg-AD mice, a transgenic model exhibiting Aβ plaques and tau tangles. SLAB51 treated mice reduced proinflammatory cytokine production (IL-1β, IFN-γ, and TNF-α) and cerebral Aβ, while simultaneously increasing SCFA production and cognitive function. However, there were no significant changes in microbial taxa, but rather an overall shift of the gut microbiota following SLAB51 treatment [76]. Another study employed the prebiotic, fructooligosaccharides (FOS), to modulate the gut microbiota in APP/PS1 mice. FOS treated mice exhibited decreased Aβ deposition, increased cognitive function and synaptic plasticity, and reversed altered gut microbiota in APP/PS1 mice to more closely resemble wildtype mice [77]. A study on the impact of high-fat diet in 3xTg-AD mice showed increased Rikenellaceae and Lachnospiraceae and decreased Bifidobacteriaceae and Lactobacillaceae in the colon when compared to normal fed 3xTg-AD mice [78]. Wang and colleagues treated 5xFAD mice with sodium oligomannate (GV-971), an algae-based drug, which remodeled the gut microbiota, and reduced M1 microglia activation and neuroinflammation. These findings suggest gut microbiota derived metabolites including the amino acids phenylalanine and isoleucine invoke neuroinflammation. In the same study, 5xFAD mice were co-housed with wildtype mice from birth to seven months. The results showed the gut microbiota of the 5xFAD and wildtype had shifted to resemble each other, and co-housed wild type mice had increased infiltrating Th1 cells in the brain, indicating the role of the gut microbiota composition in changes immune cell expression and neuroinflammation [35]. FMT treatment of APP/PS1 with wildtype mouse feces alleviated cognitive decline, Aβ accumulation, and Tau hyperphosphorylation through reversing alterations in the gut microbiota of APP/PS1 mice. To illustrate, before the treatment, APP/PS1 mice were enriched with Proteobacteria and Verrucomicrobia; however, post-treatment, the abundance of Proteobacteria and Verrucomicrobia had decreased, while Bacteroidetes increased [79].

## 7. Potential Role of the Gut Microbiome in Neuroinflammation in AD

Neuroinflammation has recently been recognized as a key feature of AD. Neuroinflammation is the body’s combined biochemical and cellular responses of all resident glial cells to injury or infection of the nervous system and neurodegenerative diseases [80]. To prevent damage to the CNS, resident immune cells recognize any disturbances to homeostasis of the brain and respond with production of cytokines and chemokines to mediate tissue damage [81]. In healthy adults, aging leads to changes in the immune system, resulting in what is often termed “inflammaging”, or chronic, low-grade inflammation due to aging [82]. Microglia increase responsiveness to inflammatory stimuli with aging, while astrocytes act to preserve neuroinflammation [83]. The main driver of age-related neuroinflammation in Alzheimer’s patients is reactive gliosis, or the activation of the glial cells of the CNS to prevent and repair tissue damage [84]. Age-related changes in microglial activation result in changes in gene expression, rendering dysmorphic microglia with morphological alterations, including abnormalities in cytoplasm and fragmented processes [85]. These changes may be related to the gut microbiome. A study in germ-free mice demonstrated increased abundance of *Desulfovibrio*, a gut bacterium associated with intestinal inflammation, after transfer of an aged mouse gut microbiota [86]. These findings suggest unique gut microbiota compositions in aging mice may be associated with chronic, low grade inflammation.

While neuroinflammation is initially a reparative mechanism to prevent damage, the neuroinflammatory response in AD patients shifts from neuroprotective to neurotoxic, damaging tissue in the CNS [51]. Neuroinflammation remains constant, with consistently high levels of cytokines and chemokines, and increased neuronal cell death [87]. This chronic inflammation is associated with shorter and fewer microglial processes, reducing mobility, thereby inhibiting their ability to survey and monitor their environment [64,88]. The cytokines produced by microgliosis and astrogliosis worsen tauopathy and drive sustained neuroinflammation [89].

### 7.1. Neuroinflammation: Microglial Activation and the Gut Microbiome

Many diseases of the CNS that are linked to the gut microbiome, such as AD, Parkinson’s Disease, Multiple Sclerosis, and Autism Spectrum Disorder, simultaneously involve dysfunctional microglia [90]. Microglia are the main immune cell in the brain, colonizing the brain early in development, and maintaining homeostasis during aging [91]. As the specialized macrophages of the CNS, microglia function as phagocytes to clear bacteria, cellular debris, and Aβ peptides [92,93]. Microglia work to monitor their microenvironment through constant extension and retraction of their highly motile processes [94]. The acute neuroinflammatory response induced by Aβ peptides is a self-limiting, neuroprotective immune response [72]. However, aging microglia are ineffective at phagocytosing the neurotoxic Aβ plaques present in AD [95]. Instead, microglia are activated by aggregated Aβ plaques, driving a chronic neuroinflammatory response [38].

Microglia receive input from not only the brain but also the GI tract via the vagus nerve [57]. When the vagus nerve senses a change in inflammation and proinflammatory cytokine production in the GI tract, afferent fibers relay this information to the brain and influence neuroinflammation [59]. However, electrical stimulation of the vagus nerve attenuates neuroinflammation induced by peripheral LPS (lipopolysaccharide), a component of the Gram-negative bacterial cell envelope that can be released upon cell lysis. In one study, C57BL/6 mice were challenged with peripheral LPS and subjected to vagus nerve stimulation (VNS). VNS in the presence of LPS resulted in an increase of IL-6, IL-1β, and TNF-α [96]. A study by Huffman and colleagues challenged C57BL6/J mice with *Escherichia coli* derived LPS to induce neuroinflammation. Percutaneous VNS stimulation reduced neuroinflammation, restored LPS-induced cognitive decline, and modulated microglial activity [97]. Another study using external, non-invasive VNS in 12-month old APP/PS1 transgenic AD mice illustrated a shift from neurodestructive to neuroprotective microglia phenotypes [64]. Taken together, these studies demonstrate that LPS is a potent stimulator of microglia via the vagus nerve, and vagal nerve stimulation may reverse neurotoxic microgliosis.

Microglia polarization is determined by a complex group of activation processes by different stimuli in the CNS, resulting in microglia polarization [98]. When an injury is present, microglia are capable of cytotoxic response and immune regulation, to resolve the damages [99]. Differentiated microglia polarize to two main phenotypes: pro-inflammatory or M1, and neuroprotective or M2 [100]. Microglia phenotype is based on their activation pathways that communicate via environmental signals to alter gene expression and cellular metabolism [101]. Classical activation by lipopolysaccharide (LPS) and IFN-γ polarize microglia cells to an M1 state, inducing production of pro-inflammatory cytokines, including TNF-α, IL-1β, IL-12 [102,103]. Alternative activation by IL-4 and IL-13 polarize microglial cells to an M2 state, inducing production of anti-inflammatory cytokines, including IL-4, IL-13, IL-10, and TGF-β [102]. Thus, microbial signals may be critical in M1/M2 polarization and therefore neurological health.

Microglia and the gut microbiota communicate via the gut microbiota–brain axis, regulating immune homeostasis. Wang and colleagues demonstrated M1 polarized neuroinflammation in 5xFAD mice is a result of abnormal production of amino acids, including phenylalanine and isoleucine, by the gut microbiota. Increased M1 activation as a result of crosstalk with Th1 cells infiltrating the CNS induced pathological neuroinflammation and cognitive decline [35]. Gut microbiota alterations may also affect microglia activation by controlling maturation and function. In this study, adult germ-free mice, lacking microbiota signaling, have distinct microglia with changes in density and morphology [57]. Furthermore, a study on critical hypertension indicated the influence of microglia dysfunction and neuroinflammation on the microbial communities in the gut. In this study, inhibition of microglia activation led to changes in the phylum Proteobacteria, suggesting that microglia activity plays a role in modulating the gut microbiota composition [104].

### 7.2. Neuroinflammation: Astrocyte Activation and Gut Microbiome

Astrocytes are specialized resident glial cells in the CNS, and responsible for protection, support of neurons, and overall homeostasis [105]. When damage occurs to the CNS, astrocytes drive an inflammatory process called astrogliosis [106]. Astrocytes function in Aβ clearance and blood brain barrier (BBB) integrity maintenance through production of cytokines and chemokines, including IL-1, IL-6, and TNF-α, to signal a pro-inflammatory innate immune response [35,107,108]. Making up about 50% of human brain mass, astrocytes play critical roles in neurodegenerative disorders and cognitive decline, including AD [109].

Neuroinflammation in AD is exacerbated by dysfunction of astrocytes, resulting in decreased BBB integrity and recruitment of immune cells from the blood. Astrocyte dysfunction can lead to deposition of Aβ peptides in the brain and results in endothelial cell damage and leakage of the blood brain barrier [110]. In one study, patients with mild cognitive impairment and early AD underwent dynamic contrast material-enhanced magnetic resonance (MR) imaging to analyze BBB leakage, demonstrating cognitive decline is directly related to severe leakage [111]. Furthermore, cytokines are able to both cross the blood brain barrier and damage the blood brain barrier by increasing permeability without entering the brain [112]. IL-1β, IL-6, and TNF-α have been shown to modify functional activity of the BBB and can be selectively transported across the BBB [113]. Aβ adhesion to microglia induces proinflammatory gene expression, producing IL-1β and TNF-α, thereby activating astrogliosis and amplifying the neuroinflammatory response. Increased proinflammatory cytokine production and neuroinflammation eventually lead to tau hyperphosphorylation and neuronal loss [114,115]. Recent studies have shown that gut microbiome impacts the function of astrocytes. The microbial product, LPS, potentiates the production of inflammatory mediators from astrocytes, leading to neurodegeneration [116]. LPS has been used in many studies to induce microgliosis, astrogliosis, and neuronal apoptosis [117,118,119]. Increases in the relative abundance of *Nitriliruptor, Youngiibacter, Burkholderia*, and *Desulfovibrio*, in children with an autism spectrum disorder, correlate with astrocyte activation [120]. Additionally, SCFA production by the gut microbiota may inhibit astrocyte activation. A study by Liu and colleagues demonstrated acetate, propionate, and butyrate, common SCFAs produced by the gut microbiota, suppress LPS and proinflammatory cytokine production through inhibition of the NF-κB pathway in vitro [121]. However, increased Lachnospiraceae, Ruminococcaceae, and Prevotellaceae in the GI tract of NLRP3-deficient mice ameliorated astrocyte dysfunction and reduced depressive-like behaviors [122]. These findings suggest astrocytes may be a potential therapeutic target for neurological disorders, including AD, associated with astrocyte activation and dysfunction.

## 8. Microbial Etiology Hypothesis in AD

Changes in gut microbiota composition and diversity during aging can drive age-associated inflammation; recent studies in *Drosophila* [123] and in murine models [124,125] suggest that alterations to the gut microbiota in late life drive increased intestinal permeability and increased systemic inflammatory markers. Since neuroinflammation is a key feature of Alzheimer’s disease (AD), understanding the aging gut microbiome is critical to understanding AD progression. Mechanistically, bacteria in the GI tract can produce a significant amount of amyloids (aggregated, insoluble proteins exhibiting β-pleated sheet structures) can contribute to Aβ plaque formation and increase the risk for AD development [126,127,128]. Microbiome-derived functional amyloids have been described in the multiple microbial taxa, including *Saccharomyces cerevisiae*, and broadly in the family Enterobacteriaceae. *Escherichia coli* extracellular amyloids known as curli fibers help to facilitate attachment to surfaces and protection against host defenses [129,130,131,132]. Both curli fibers from *E. coli* and sup35 from *S. cerevisiae* were capable of enhancing amyloid aggregation and amyloidosis in a murine model of experimental amyloidosis, indicating that microbial-derived amyloids may have prion-like properties [133]. In another study, amyloids derived from three species of Gram-negative bacteria (*E. coli, Salmonella typhimurium*, and *Citrobacter koseri* could induce polymerization of amyloid aggregates across species in vitro, again demonstrating the potential for microbiome-derived amyloids to contribute to amyloidosis in AD [134]. Microbial amyloids may also enhance the immune response to endogenous amyloids, contributing to neuroinflammation. Microbial amyloids are sensed by Toll-like receptor 2 (TLR2), which can induce expression of many of the neuropathological features observed in AD, discussed above. Curli from *S. typhimurium* was sufficient to induce IL-17A, IL-22, and IL-6 in the intestinal epithelium, and these responses were blunted in TLR-2 deficient mice [135]. However, these findings must be placed into the context of a healthy or dysbiotic microbiome; another study found that commensal-derived amyloids may help to maintain the epithelial barrier and induce anti-inflammatory IL-10 expression in the gut [136]. The altered microbiome composition observed in human and preclinical models may lead to impaired epithelial barrier, allowing microbiome-derived amyloids to translocate, cross-seed with endogenous peptides, or activate a neuropathological immune response.

The endotoxin hypothesis of neurodegeneration states that the endotoxin, or LPS, found in the outer membrane in all Gram-negative bacteria, crosses the blood brain barrier to induce neuroinflammation and neurodegeneration. The proposed pathway begins with a GI infection, or perhaps an increase in absolute abundance of a Gram-negative gut microbes, results in increased circulating endotoxins, which cross the BBB, leading to chronic neuroinflammation and finally, neurodegeneration [137,138]. Dysbiosis of the gut microbiota resulting in enrichment of Gram-negative bacteria in the gut microbiota is associated with increased LPS-driven inflammation [139]. Notably, *Bacteroides fragilis*, a known LPS-producing gut bacterium, is hypothesized to contribute to inflammatory signaling in AD patients via the NF-κB pathway [140].

**Figure 1:**
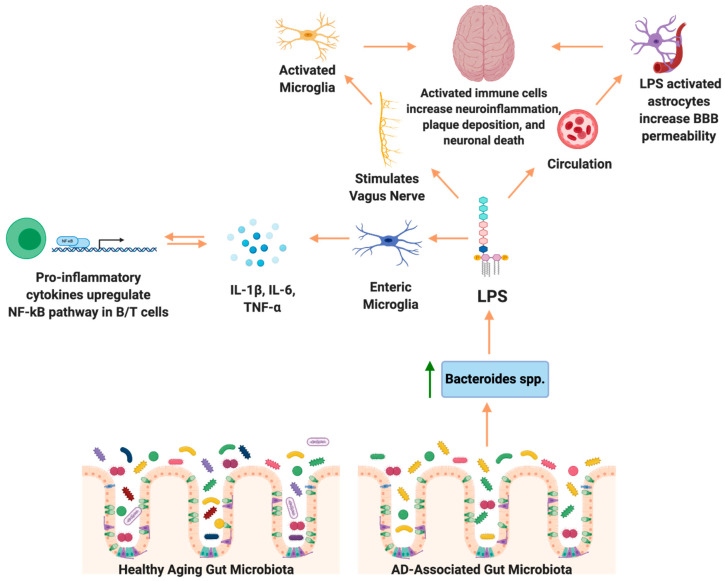
Proposed mechanism of lipopolysaccharide (LPS)-induced neuropathologies in Alzheimer’s disease (AD). AD-associated microbiome composition has increased abundance of lipopolysaccharide (LPS) producing bacteria, including *Bacteroides*. LPS stimulates enteric microglia to produce Interleukin (IL)-1β, IL-6, and Tumor necrosis factor (TNF)-α, which induces the nuclear factor kappa-B (NF-κB) pathway, therefore upregulating expression of proinflammatory cytokines. Similarly, LPS stimulates the vagus nerve, activating microglia in the brain, thereby promoting neuroinflammation. LPS is able to translocate from the gastrointestinal tract to the circulatory system and stimulate astrocytes. The reduction in blood brain barrier (BBB) integrity allows LPS to cross the BBB and further promote inflammation in the brain.

Peripheral LPS may contribute to neuroinflammation and Aβ plaque deposition in the brain (summarized in Figure 1). LPS in the gut activates the enteric nervous system, and can stimulate production of the proinflammatory cytokines TNF-α, IL-1β, and IL-6, which are secreted in the gut and can travel to the periphery [141]. More specifically, activation of the ENS leads to TNF-α induced inhibition and production of IL-6 in the GI tract or ENS cells in vitro, a known inflammatory cytokine in AD [142]. Inflammation of the GI tract increased intestinal permeability to recruit immune cells from circulation, however also allowing inflammatory bacterial components to cross the barrier into the peripheral circulatory system, eliciting systemic inflammation [87]. Pathogenic microbial metabolites, proinflammatory cytokines and other molecules produced by the gut microbiota, including LPS, are associated with neuronal death and neuroinflammation [143]. TLR-4, a known LPS receptor, is localized on microglia, astrocytes, and neurons, and plays a role in both cell survival and death [144]. The activation of TLR-4 on microglia and other neural cells leads to activation of the NF-κB pathway, thereby increasing the production of cytokines and Aβ deposition. Additionally, TLR-4 is expressed in vagal afferent fibers, which allow the vagus nerve to sense LPS and activate the brain [59]. In rats, IP injection of LPS resulted in increased expression of IL-6, IL-1β, and TNF-α in the brain, and LPS co-associated Aβ plaque deposition [145]. The role of LPS in the development of AD is reviewed in [146].

A considerable amount of research points towards an infectious etiology of AD. Inflammation is an essential process in healing damaged tissue and fighting off infection, however, chronic inflammation leads to permanent damage. Studies have noted bacteria, viruses, protozoa, and fungi as potential agents in systemic infections related to AD [147]. Injection of *Helicobacter pylori*, an opportunistic pathogen of the GI tract, induced memory impairment and Aβ plaque deposition through increased gene expression of presenilin-1 in rats and in vitro. In the same study, injection of *Escherichia coli*, another common gut pathogen, demonstrated no change in cognitive impairment or plaque deposition [148]. Furthermore, in another study, different regions of the brain in AD patients, including the frontal cortex and hippocampus, had evidence of *Candida* spp. cells and hyphae, while control patients showed no signs of fungal cells or hyphae, providing intriguing evidence for existing fungal infections in AD brains [149]. Moreover, there is evidence to suggest fungal infections in the brain can be accompanied by bacterial infections, demonstrating polymicrobial infections of the brain in AD may contribute to pathogenesis and neuroinflammation [150]. Taken together, these studies suggest translocation of microbes into the brain and chronic microbial infection may play a role in neuroinflammation and AD neuropathologies.

## 9. Potential for Microbiome-Based Therapeutics

The gut microbiota–brain axis remains a target for future therapeutics for AD. Immunotherapy [151] and gut microbiota-targeted therapy [17] are among the many potential therapeutic targets for AD. Immunotherapy targets include the anti-Aβ and anti-tau target antibodies, Aβ vaccines, and cytokine inhibition [151,152,153]. Passive immunization of naturally occurring autoantibodies injected into APPSwe mice, a single transgenic model for amyloidosis, reduced toxic Aβ oligomers, and improved cognitive function [154]. 3xTg-AD mice immunized with active, full length DNA Aβ42 demonstrated reduced plaque deposition and tauopathy [155]. LPS-treated 3xTg-AD mice injected with TNF inhibitor XENP345, a TNF cytokine inhibitor, reduced accumulation of 6E10 immunoactive protein, which contains amyloid precursor protein fragments, therefore preventing pre-amyloidosis pathologies [156]. Neuroimmune therapeutics targeting reduction of Aβ plaques and neurofibrillary tangles continue to evolve as understanding of disease pathologies becomes more clear. Due to the complexities involved in disease progression, combinatorial immunotherapy may hold promise to a future of immunotherapeutics for AD [157].

Potential gut microbiota target therapies include prebiotic/probiotic supplementation and fecal microbiota transplantation to restore gut microbiota to a diverse, healthy microenvironment [158]. In 3xTg-AD mice, oral probiotic supplementation reduced Aβ burden and hyperphosphorylated tau aggregates through gut microbiota manipulation [159]. A study using E4FAD mice, a model for late onset AD, demonstrated that supplementation with the prebiotic inulin increased SCFA’s and reduced neuroinflammation [160]. Early probiotic trials in patients with AD or mild cognitive impairment remain promising with results showing improved cognitive function, suggesting the involvement of the gut microbiota brain axis [161,162]. Despite the numerous studies on transgenic mice modeling AD pathologies, few translational studies in AD patients have been published. A case study of an 82 year old man with AD followed the patient over a six month period following an FMT treatment for a *Clostridioides difficile* infection. The patient showed significant improvement on his Mini-Mental State Examination and scored in the normal cognition range at two and six months post treatment. However, this was a case study, and a clinical trial of the effects of FMT on AD patients through modulation of the gut microbiota–brain axis is warranted [163].

Another method of gut microbiota targeted therapy is through dietary intervention. Numerous epidemiological evidences have demonstrated a link between diet, gut microbiota, and human health [164,165,166]. However, only recently have the mechanisms linking diet and human health been uncovered. Diet and lifestyle are closely related to cardiovascular and metabolic diseases, which are connected to neurological disorders. For example, insulin resistance and high plasma homocysteine levels, which are indicators of metabolic syndrome (Mets), play a major role in cerebral physiology and morphology, amyloid-β deposition, and neurological plaque accumulation [167]. In addition, several studies have demonstrated that healthy diet patterns such as Mediterranean diet (MeDi), dietary approach to stop hypertension (DASH), and Mediterranean-DASH diet intervention for neurodegenerative delay (MIND) are effective in reducing dementia and AD risk [168,169,170,171]. Brain glucose uptake is impaired in AD, possibly due to a loss of synapses and neuronal dysfunction [172]. If deprived of glucose, the primary energy source, the brain can switch to utilization of ketones (specifically β-hydroxybutyrate (β-HB) and acetoacetate (AcAc); [173,174]). Interestingly, brain ketone uptake remains normal in AD patients [175,176]. Thus, several studies and review articles have assessed the potentially beneficial effect of a ketogenic diet in AD [177,178,179,180]. Indeed, a systematic review of randomized clinical trials evaluating ketogenic and non-ketogenic dietary interventions in participants with mild cognitive impairment (MCI) and AD showed promising results from the studies included, despite heterogeneity in study design, dietary compliance, and participants [181].

The gut microbiome is greatly affected by these dietary interventions. Changes in the composition of the intestinal microbiota caused by diet affect the various metabolites they produce, and these changes can regulate the host immune response in a beneficial way and maintain the health of the nervous system. For example, a ketogenic diet increased relative abundances of potentially beneficial bacteria (*Akkermansia* and *Lactobacillus*) compared to mice fed a control diet. This correlated with enhanced neurovascular function, and amyloid-β clearance which was associated with reduction in mTOR expression and increased endothelial nitric oxide synthase (eNOS) protein expression [182]. In a study of patients with mild cognitive impairment, gut microbiota composition is associated with cognitive impairment. A modified Mediterranean-ketogenic diet (MMKD) regulated gut microbiome composition and production of metabolites such as SCFA, which were associated with improvement of AD symptoms [183,184]. These studies suggest the modification of gut microbiome through dietary intervention can be a strong candidate for future AD therapy. Future studies focused on the gut microbiota–brain axis as a therapeutic utilizing a multi-omics approach, which is crucial for uncovering the role of the gut microbiota–brain axis in AD and the development of therapeutics.

## 10. Limitations of Current Research

Current research on the gut microbiota–brain axis in Alzheimer’s disease is based mostly on compositional microbiome data using 16S rRNA marker gene sequencing. Few studies have evaluated the role of fungal microbiota in the gut, though recent work has demonstrated a critical role of fungal communities in health and development of peripheral organs through sensing of fungi by gut-resident immune cells, such as mononuclear phagocytes [185]. While this approach has laid the foundation for the field, the use of transcriptomics, metagenomics, and metabolomics will be necessary to uncover the complex set of mechanisms involved in the gut microbiota–brain axis. There are a limited number of studies in human AD patients, as highlighted by the minimal studies cited in this review, and the studies in human AD to date have been observational. Thus, we cannot yet determine the directionality of the observed changes in the gut microbiota in AD, e.g., whether gut microbiome dysbiosis is due to altered diet, medication use, or stress in individuals with AD. However, preclinical murine models of AD pathologies demonstrate altered gut microbiota prior to emergence of amyloidosis, tauopathy, or neuroinflammation, which provides some, but limited, evidence that changes in the gut microbiome may contribute to AD progression. Future longitudinal, cross-sectional, and interventional studies in AD patients are warranted to study key changes in the gut microbiota composition, mechanistic pathways, and possible therapeutics.

In a broader sense, treatment of disease through modification of the gut microbiome is extremely challenging due to the myriad of factors that shape microbiome composition and function. Development of the gut microbiome begins at birth, and rapidly changes during the first three years of life [186]. The gut microbiome is influenced by host genetics, mode of delivery, diets, surrounding environment, and lifestyles of individuals, and even psychological factors such as stress and anxiety [187,188,189,190]. For this reason, it is difficult to accurately distinguish which gut microbiota contributes to the onset or progression of Alzheimer’s disease, and even if such gut microbiota is found, it is difficult to modify and maintain changes to the gut microbiome. Patients with Alzheimer’s disease experience a range of dietary, medical, and stress-related changes as dementia progresses, which may influence the composition of the gut microbiome independent of disease pathologies. For future studies, it is important to have accurate understandings of how modifying the gut microbiome can contribute to health status of AD patients, and to determine how to maintain a healthy microbiome using a combination of rationally-developed probiotics (live microorganisms that confer a health benefit), prebiotics (non-digestible dietary ingredients that promote the growth of beneficial microorganisms), or dietary interventions.

## 11. Conclusions

Thus far, we have summarized the role of the gut microbiota–brain axis as an integral part of disease pathogenesis. However, this complex system employs a myriad of mechanisms working together in the development of AD. While there are many moving factors in AD pathophysiology, identifying key pathways and mechanisms involved in progression is crucial in order to develop novel therapeutic targets. Growing evidence supports bidirectional communication between the CNS and the gut microbiota; studies demonstrate that the microbiota shapes AD pathologies and neuroinflammation, and AD pathogenesis modulates the gut microbiota composition. The gut microbiota–brain axis modulates key processes, including immune cell maturation, SCFA, LPS, and cytokine production, permeability of the gut epithelium and BBB, and gut microbiota diversity. Gut microbiota production of SCFA is reduced in AD thereby inducing the NF-κB pathway and increasing production of proinflammatory cytokines, including IL-1β, IL-6, and TNF-α [191]. These cytokines are able to modulate the BBB permeability and translocate to the brain [113]. Once in the brain, proinflammatory cytokines potentiate Aβ deposition, tau hyperphosphorylation, microgliosis, and astrogliosis, increasing the severity of AD pathologies. Further studies are warranted to identify the directionality of observed changes in the microbiota, mechanisms, and therapeutic strategies with these novel targets. In particular, focus on elucidating the interactions between the gut microbiota communities and cytokines and their role in neuroinflammation and disease pathologies will lead to a better understanding of disease mechanisms. Studies with a multi-omics approach in both animal models, carefully selected human cohorts, and in vitro mechanistic studies will uncover the underlying mechanisms of the gut microbiota–brain axis and its impact on AD, dementia, and other neurological diseases. Focus on neuroinflammation and cytokines in the gut microbiota–brain axis will elucidate the role of chronic inflammation in AD and other inflammatory diseases.

## Figures and Tables

**Table 1:** Alterations in gut microbiota composition in humans and transgenic mice without intervention.

Species	Increased Abundance	Decreased Abundance	Citation
Human	g.*Blautia*, g.*Alistipes*, g.*Bacteroides*	g.*Turicibacter*, g.*Clostridium*, g.*Dialister*	Vogt et al. [66]
Human	g.*Gammaproteobacteria*, g.*Enterobacteriales* and f.*Enterobacteriaceae*	g.*Ruminococcus* f.Clostridiaceae, f.Lachnospiraceae	Liu et al. [67]
Human	g.*Ruminococcus*, g.*Subdoligranulum*	f.Lachnospiraceae, g.*Lachnoclostridium*, g.*Bacteroides*	Zhuang [68]
Human	g.*Bacteroides*, g.*Alistipes*, g.*Odoribacter*	g.*Butyrivibrio*, g.*Eubacterium*, g.*Lachnoclostridium*,	Haran et al. [69]
APP/PS1	g.*Desulfovibrio*, g.*Akkermansia*, f.Lachnospiraceae, g.*Ruminococcus*	g.*Alistiples*	Chen et al. [72]

**Table 2:** Alterations in gut microbiota composition in transgenic mice after the intervention.

Species	Increased Abundance	Decreased Abundance	Intervention	Citation
3xTg-AD	g.*Bifidobacterium*	o.Campylobacterales	SLAB51 probiotic	Bonfili et al. [76]
APP/PS1	g.*Lactobacillus*	g.*Helicobacter*	FOS supplement	Sun et al. [77]
3xTg-AD	f. Rikenellaceae, f.Lachnospiraceae, f.Enterococcaceae and f.S24.7	g.*Bifidobacterium*, g.*Lactobacillus*	Fatty diet	Sanguinetti et al. [78]
